# Development of a Bilayer Tablet by Fused Deposition Modeling as a Sustained-Release Drug Delivery System

**DOI:** 10.3390/ph16091321

**Published:** 2023-09-19

**Authors:** Andrea Gabriela Crișan, Alina Porfire, Sonia Iurian, Lucia Maria Rus, Raluca Lucăcel Ciceo, Alexandru Turza, Ioan Tomuță

**Affiliations:** 1 Department of Pharmaceutical Technology and Biopharmacy, Faculty of Pharmacy, “Iuliu Hațieganu” University of Medicine and Pharmacy, 41 Victor Babeș Street, 400012 Cluj-Napoca, Romania; crisan.andrea@umfcluj.ro (A.G.C.); sonia.iurian@umfcluj.ro (S.I.); tomutaioan@umfcluj.ro (I.T.); 2 Department of Pharmaceutical Analysis, Faculty of Pharmacy, “Iuliu Hațieganu” University of Medicine and Pharmacy, Louis Pasteur Street 6, 400349 Cluj-Napoca, Romania; lucia.rus@umfcluj.ro; 3 Faculty of Physics, Babeș-Bolyai University, 400084 Cluj-Napoca, Romania; raluca.lucacel@ubbcluj.ro; 4 Interdisciplinary Research Institute on Bio-Nano-Science, Babeș-Bolyai University, 400271 Cluj-Napoca, Romania; 5 National Institute for Research and Development of Isotopic and Molecular Technologies, 67-103 Donath Street, 400293 Cluj-Napoca, Romania; alexandru.turza@itim-cj.ro

**Keywords:** hot-melt extrusion, 3D printing, fused deposition modeling, sustained release, polyvinyl alcohol, customization

## Abstract

Three-dimensional printing by fused deposition modeling (FDM) coupled with hot-melt extrusion (HME) is a point of convergence of research efforts directed toward the development of personalized dosage forms. In addition to the customization in terms of shapes, sizes, or delivered drug doses, the modulation of drug release profiles is crucial to ensure the superior efficacy and safety of modern 3D-printed medications compared to those of conventional ones. Our work aims to solidify the groundwork for the preparation of 3D-printed tablets that ensure the sustained release of diclofenac sodium. Specifically, we achieved the fast release of a diclofenac sodium dose to allow for the prompt onset of its pharmacological effect, further sustaining by the slow release of another dose to maintain the effect over a prolonged timeframe. In this regard, proper formulation and design strategies (a honeycomb structure for the immediate-release layer and a completely filled structure for the sustained-release layer) were applied. Secondarily, the potential of polyvinyl alcohol to function as a multifaceted polymeric matrix for both the immediate and slow-release layers was explored, with the objective of promoting the real-life applicability of the technique by downsizing the number of materials required to obtain versatile pharmaceutical products. The present study is a step forward in the translation of HME-FDM-3DP into a pharmaceutical manufacturing methodology.

## 1. Introduction

The administration of medications via the oral route is the most convenient alternative since it is simple, affordable, and non-invasive [1,2], thereby ensuring adequate patient compliance. However, adherence to treatment plans often decreases when the drug is included in immediate release (IR) formulations that require frequent administration to maintain therapeutic concentration levels. Moreover, a greater risk of adverse events or falling outside the therapeutic window are consequences of multiple administration regimens due to the potential fluctuation of the plasmatic concentrations above or below the therapeutic levels [3,4].

Oral sustained-release formulations attain steady plasmatic drug concentrations within the therapeutic interval for a prolonged timeframe and therefore address the shortcomings of traditional IR products [5]. By reducing the number of required administrations, maintaining the plasmatic concentrations within the therapeutic range, and consequently avoiding distressing adverse events, sustained-release formulations can ensure a better patient compliance.

The conventional manufacturing techniques applied for the preparation of sustained-release tablets involve complex and rigid processes optimized to deliver products with invariable doses, active pharmaceutical ingredient (API) combinations, and release profiles [6]. Moreover, their disadvantages, which include elaborate and time-consuming fabrication steps or expensive equipment, urge us to pursue alternative methods that allow the simple preparation of tailored dosage forms in terms of API combinations, doses, and release profiles [7].

The versatility and flexibility of fused deposition modeling 3D printing (FDM-3DP) brought this technology into the pharmaceutical landscape. A core driver is the opportunity to harness its potential for the on-demand fabrication of patient-tailored drug delivery systems. Thus, the preparation of dosage forms with customizable drug release patterns is among the prerequisites that need to be evidenced to support the translation of the technique into clinical and pharmaceutical settings. Consequently, the prospect of fabricating sustained-release tablets via FDM-3DP is of utmost importance. Moreover, since a highly sought-after application of FDM-3DP is the manufacturing of small batches of tailored drug products in community pharmacies and hospitals, the simplification of the process could enhance the transferability to these settings. Accordingly, the possibility of using the same material (i.e., polyvinyl alcohol—PVA) to fabricate drug-loaded filaments that could be employed to prepare end products with a range of customizable properties (i.e., drug release profiles) would make the technique easier to implement.

The fabrication methodology by FDM-3DP involves the utilization of filaments based on thermoplastic polymers as starting materials for the 3D printer. Concretely, during the printing process, the filament is pushed towards the heated compartment of the printing head by a system of feeding gears, where the material is liquefied and forced through a nozzle by the following fresh feedstock filament that acts as a piston. The melted material is deposited on a printing platform in successive layers according to the object’s architecture generated in a 3D design software [8,9].

In the case of pharmaceutical FDM-3DP, the drug is typically included in the feedstock filament. Earlier approaches involved loading the APIs by soaking commercially available filaments into solutions of the drug of interest. However, drawbacks, such as the limited drug-loading capacity that (to our knowledge) reached a maximum of 4% [10], time-consuming steps, and the loss of a considerable quantity of the APIs in the residual solution [11], fueled the change in direction towards the fabrication of drug-containing filaments via hot-melt extrusion (HME). The method implies processing a physical mixture of the APIs and appropriate excipients at elevated temperatures. This results in obtaining a melted mass that is ultimately pushed through a nozzle and solidifies at ambient temperature into a filament shape.

Polyvinyl alcohol (PVA) is among the preferred polymeric materials involved in the formulation and preparation of FDM-3D-printed pharmaceutical dosage forms. In addition to its excellent thermoplasticity, which is a prerequisite for the feasibility of the fabrication technique, it is also biocompatible, biodegradable, and generally recognized as safe (GRAS), with an extensive safety history related to its use for drug delivery purposes [12,13]. In addition, its versatility was evidenced by studies that reported the suitability of PVA in the formulation of FDM-3D-printed dosage forms that ensured drug release patterns ranging from immediate to modified release. Uboldi et al. evaluated different polymers to develop 3D-printed immediate-release tablets containing timapiprant. The results highlighted PVA as an adequate alternative due to the superior printability of the filaments and the feasibility of the PVA-based timapiprant-loaded printlets that comply with the dissolution criteria outlined for immediate-release formulations [14]. The results obtained by Skowyra et al. demonstrated the appropriateness of PVA to function as a modified-release matrix by preparing extended-release FDM 3D-printed tablets that prolonged the API dissolution to up to 24 h [15].

The aim of the present study is to formulate and develop an FDM-3D-printed tablet that ensures sustained drug release. More precisely, the scope is to attain the rapid release of an API dose that could ensure the fast onset of the pharmacological effect, in association with the slow release of another API dose, which could sustain it. The considered methodologies to accomplish the defined objectives involved formulation and design strategies. Namely, an original bilayer tablet design was created, with each layer being characterized by distinct drug-release rates. A honeycomb structure was considered for the IR layer, based on the results obtained in our previous work [16], while a completely filled structure was designed to ensure prolonged release through a low surface to volume ratio. In addition, the fabrication methodology involved dual printing since two different filaments were considered for the preparation of the two layers. Concretely, the filament optimized by us in a previous study (Fil-PVA-P-D50, further codified as Fil-IR-PVA) [16] was used for printing the IR layer, while filaments with a high polymeric content based on PVA or the combination of PVA and Kollidon^®^ SR in different ratios were evaluated for the preparation of the SR layer.

## 2. Results and Discussions

### 2.1. Formulation and Design Strategies

The objective of this work was to prepare FDM-3D-printed sustained-release drug delivery systems. More concretely, we desired to achieve the fast release of an initial API dose, along with the slow dissolution of another over an extended timeframe. Therefore, both formulation and design strategies were considered to attain the target characteristics. First, a bilayer tablet architecture was created to delimit two distinct sections: the IR layer, consisting of a honeycomb pattern and explored in our previous study [16], and the SR layer, which corresponds to a completely filled (100% infill) tablet structure. The honeycomb pattern was identified in our previous study [16] as a suitable design to achieve the fast release of the APIs due to its high surface to volume ratio, which is a prerequisite for a rapid dissolution process. The filament loaded with 50% *w*/*w* DCNa (Fil-IR-PVA), which was developed within our previous work [16], was used for printing the IR layer. In addition to the use of the honeycomb design of the immediate-release layer, which favors the contact with the dissolution medium, a formulation strategy was used to increase the release rate, i.e., a high concentration of the active substance (50%) incorporated in a low-density polymer matrix (40%), with the help of a plasticizer (KTPS 10%). On the other hand, the design rationale for the SR layer involved ensuring a low porosity and surface area to volume ratio since the slow erosion of the polymeric structure with the decelerated release of the APIs were the target objectives for this layer. For the FDM-3DP of the SR layer, different filaments with the compositions presented in Table 1 were considered. The drug content (10% *w*/*w*) of each formulation was low, in order to ensure a high polymer concentration, since the dissolution of the APIs is conditioned by the erosion of the polymer and previous works highlighted that greater polymer fractions negatively impact the API release rates [17].

### 2.2. Solid State Evaluation

The stability of the materials during filament fabrication and tablet preparation is a critical concern since high processing temperatures are a prerequisite of both HME and FDM-3DP technologies. Thus, TGA investigations were conducted for the individual components, as well as for the physical mixtures, filaments, and tablets, and the results are displayed in the Supplementary Materials (Figures S1–S3). The TGA curves of the formulations relying solely on PVA as a matrix-forming polymer are shown in Figure S1a (Supplementary Materials). The debut of a progressive weight loss was observed at approximately 210 °C for both the physical mixture and filament samples. In Figures S1b, S2 and S3 (Supplementary Materials), the TGA profiles of the samples associated with the formulations relying on the combination of PVA and Kollidon^®^ SR as a polymeric matrix are displayed. The physical mixtures followed the pattern described for the samples consisting of PVA exclusively as a polymeric component. In contrast, the filament samples revealed an additional minor weight loss step (~3% *w*/*w*) that started at about 64 °C and was assigned to the presence of water. The filament used for the fabrication of the IR layer, namely, Fil-IR-PVA, along with the individual components of the formulation and the prepared tablets were characterized within our previous work by applying the same analysis protocol. The examinations revealed that the APIs are stable at the employed working temperatures (i.e., 175 °C for HME and 185–190 °C for FDM-3DP), and no significant degradation of other components occurs during thermal processing [16].

The DSC curves of DCNa, sorbitol, PVA, PM-SR-PVA, Fil-SR-PVA, and Tab-SR-PVA are displayed in Figure S4a (Supplementary Materials). From the examination of these curves, it is observable that the API exhibits its characteristic melting endotherm at approximately 287 °C, which is immediately followed by the exotherm peak that signals the decomposition process [18]. Other melting endotherms are visible in the case of sorbitol at approximately 99 °C and PVA at 191 °C [16,19]. These characteristic endotherms of the individual components are also noticeable in the thermogram of the physical mixture, except for the one corresponding to the API’s melting phenomenon, which denotes the complete solubility of the drug in the molten polymer and the formation of an amorphous solid dispersion. The melting endotherm of sorbitol is no longer visible in the DSC curves of Fil-SR-PVA and Tab-SR-PVA, which indicates that the excipient fulfilled its plasticizing function and is completely miscible with the polymer. Figures S4b, S5 and S6 (Supplementary Materials) show the DSC thermograms associated with the formulations that contain PVA and Kollidon^®^ SR as matrix-forming materials. In a similar manner to the samples lacking Kollidon^®^ SR, the endothermic peak corresponding to the melting point of DCNa is not visible in the thermograms obtained by analyzing the physical mixture, filament, and tablet samples due to the solubilization of the drug in the polymeric carrier and the development of an amorphous solid dispersion [20]. Kollidon^®^ SR is a copolymer consisting of polyvinyl acetate linked to polyvinylpyrrolidone and, accordingly, exhibits two glass transition temperatures (~30 °C and 128–180 °C). The variability of the second glass transition temperature is a consequence of inconsistent moisture content, residual monomers, and molecular masses [21]. Therefore, the slight shift of the peaks generated by the presence of Kollidon^®^ SR in the filament and tablet samples compared to those acquired by analyzing the physical mixture is most likely caused by the alteration of the moisture content following thermal processing. Regarding the DSC characterization of the IR layer, the associated samples were characterized in our previous work [16]. As opposed to the filaments loaded with 10% *w*/*w* DCNa, the peak related to the melting phenomenon of the drug was still visible in the thermograms recorded for the physical mixture, filament, and tablet samples containing 50% *w*/*w* DCNa, which indicates that the API mainly remained in its crystalline form [16].

**Figure 1:**
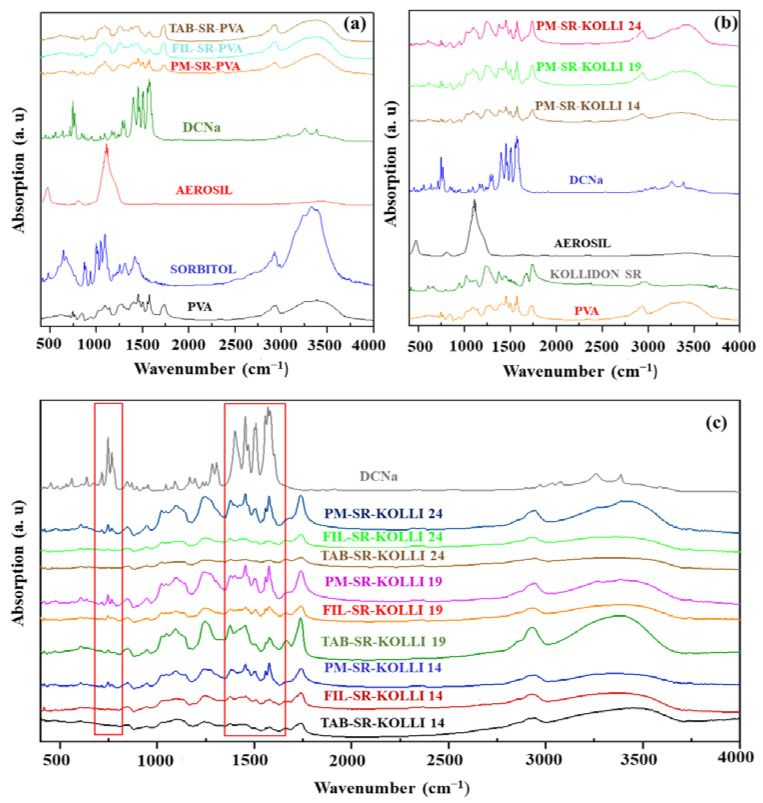
FTIR spectra of the raw materials and polymer preparations: (**a**)—formulation based solely on PVA as the matrix-forming material; (**b**,**c**)—formulations based on the combination of PVA and Kolli SR in different ratios as polymeric structures.

FTIR investigations were employed to obtain complementary data that might indicate physicochemical transformations or potential interactions among the materials, particularly following thermal processing. The results are displayed in Figure 1. The pure DCNa presented characteristic absorption bands generated by the asymmetrical and symmetrical stretching vibrations of the carboxylate groups at 1454 cm^−1^ and 1580 cm^−1^, respectively. The vibration band visible at 3375 cm^−1^ corresponds to the vibrations of the secondary amino group [22,23], whereas the peak observed at 747 cm^−1^ describes the C–Cl stretching [24]. Major absorption bands in the PVA spectrum are noticeable at 1085 cm^−1^, denoting the C–O bending vibration; 1243 cm^−1^, which is characteristic of the O–H bending vibration; 1420 cm^−1^ for the C–H bending vibration; 2910 cm^−1^ for C–H stretching; and 3287 cm^−1^ for O–H stretching. The spectrum of sorbitol exhibited a large peak starting from 3000 cm^−1^ to 3500 cm^−1^ due to the presence of numerous hydroxyl functions [25]. The spectrum of Kolli SR presents characteristic bands for C=O vibrations at 1742 cm^−1^, C=O stretching and N–H bending at 1672 cm^−1^, and C–CO–C stretching and bending at 1238 cm^−1^ [26].

The bands obtained for the raw DCNa were also expected to be noticeable in the spectra of the physical mixtures, namely, PM-SR-PVA, PM-SR-KOLLI 14, PM-SR-KOLLI 19, and PM-SR-KOLLI 24. Presumably, the low drug loading (10% *w*/*w*) and the presence of absorption bands generated by other constituents of the formulations, which overlapped the regions of interest, impaired the possibility of visualizing the characteristic bands for DCNa in both physical mixtures, filaments, and tablets, as visible in Figure 1. Thus, these limitations imposed the need for further investigations.

The FTIR examinations conducted in our previous study on samples related to the IR layer of the tablets revealed that, by processing at high temperatures, hydrogen bonds are established between the API and the polymer, a finding in accordance with the literature [25]. We also concluded that the peak-splitting phenomenon identified during the DSC investigations was allegedly caused by the physical interactions between DCNa and KTPGS [16].

**Figure 2:**
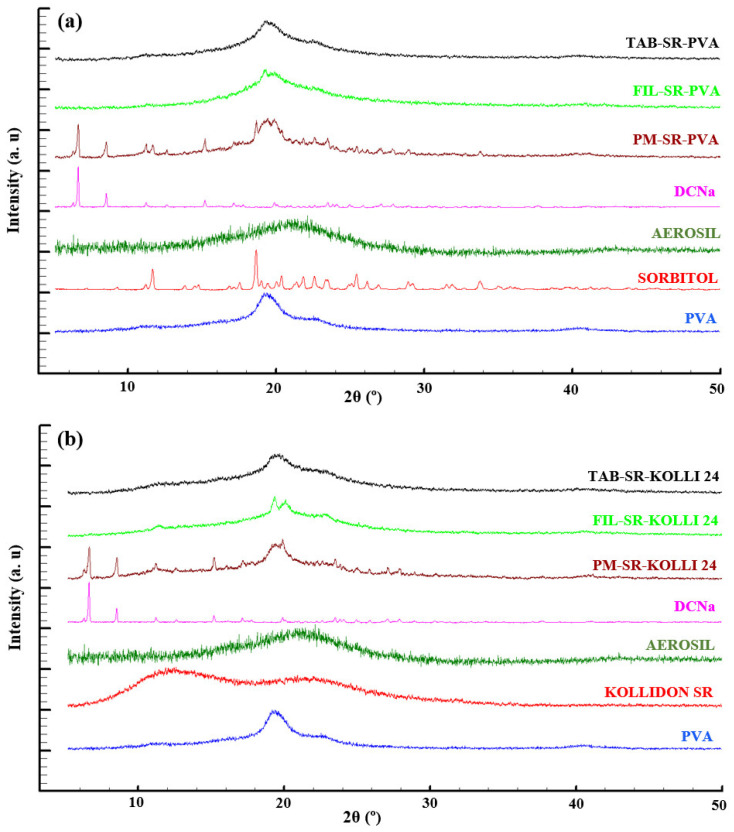
XRD diffractograms of materials, physical mixtures, filaments, and tablets consisting of (**a**)—PVA or (**b**)—the combination of PVA and Kolli SR as the matrix-forming polymers.

The diffractograms recorded by analyzing the raw materials, physical mixtures, filament, and tablet fragment samples associated with the SR layer are displayed in Figure 2. The first graphic (Figure 2a) depicts the patterns of the formulation relying exclusively on PVA as a polymeric component, while the second one (Figure 2b) depicts diffractograms obtained for the formulation based on PVA and 24% *w*/*w* Kolli SR, and it is representative of all the preparations based on the combination of PVA and different ratios of Kolli SR as the polymeric matrix, as supported by the results presented in the Supplementary Materials (Figure S7). By examining the diffraction patterns obtained for the physical mixtures (PM-SR-PVA and PM-SR-KOLLI 24), it is observable that they exhibit the characteristic peaks of the components, including those specific to crystalline DCNa. In contrast, the diffractograms of the filament and tablet samples lack these distinctive sharp peaks, which denote a conversion of the crystalline API to its amorphous form during the thermal processing of all formulations [27,28]. The findings are in complete agreement with the results obtained via DSC. Additionally, the observations are supported by the data reported by Okwuosa et al. regarding the amorphous state of DCNa included in 20% *w*/*w* drug-loaded PVP-based filaments [29]. In contrast, the investigations conducted in a previous study on samples associated with high drug loading (50% *w*/*w* DCNa) and implicitly a limited polymeric fraction (40% *w*/*w*) showed that, overall, the drug keeps its stable crystalline state in spite of being subjected to two thermal processes [16].

### 2.3. Mechanical Characterization of the Filaments

The proper mechanical properties of the hot-melt-extruded filaments are critical to ensure the feasibility of the printing process. The first requirement of a printable filament is a minimal extent of brittleness in order to avoid breakage while being subjected to the forces exercised by the feeding gears [30]. A second prerequisite is adequate flexibility, which allows for a certain degree of bending within the feeding system but still enables the filament to function as a piston, forcing the movement of the molten mass toward and through the nozzle [31,32]. Equally essential is the stiffness factor, since the filament must preserve its integrity under the action of the gear teeth, which could cause scratching, surface damage [33], and ultimately, printing failure [16]. Therefore, the proper mechanical characterization of the filaments and a deeper insight into the influence of the variables over the filament properties that ultimately determine their printability are essential to avoid the current time-consuming post-fabrication trial and error approach of evaluating the feasibility of the printing process.

**Figure 3:**
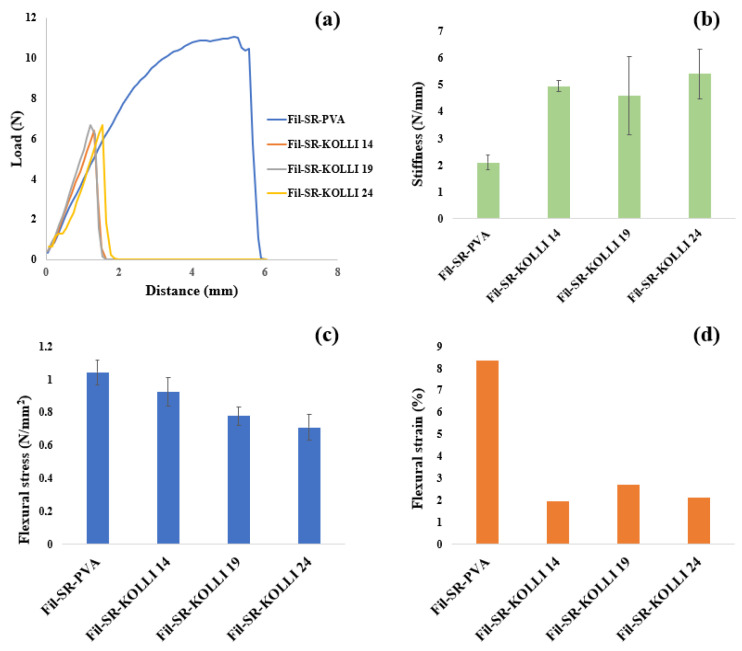
(**a**)—Load–distance curves obtained via 3-point bending test (a representative curve was selected for each formulation); (**b**)—stiffness results; (**c**)—mechanical characterization by means of flexural stress; (**d**)—mechanical characterization by means of flexural strain.

The load–distance curves based on the data recorded within the 3PB test are displayed in Figure 3a. It can be observed that the mechanical strength of the filaments reflected by the load values decreased significantly by including Kolli SR in the formulations. Additionally, a negative impact of the presence of the hydrophobic polymer on the flexibility of the filaments is indicated by the decreased breaking distances recorded for these formulations compared with values recorded for Fil-SR-PVA. This conclusion is also supported by the calculated flexural stress values displayed in Figure 3c. Similarly, a negative effect of the presence of Kolli SR in the formulations was identified in terms of the flexural strains calculated and displayed in Figure 3d. On the other hand, the hydrophobic polymer augmented the stiffness of the hot-melt-extruded filaments, according to the data shown in Figure 3b. However, despite the variability in terms of the mechanical properties created by the qualitative and quantitative adjustments of the polymeric fraction of the formulations, all four types of filaments presented adequate printability and were further employed in the fabrication of the SR layer of the bilayer tablets.

### 2.4. Physical Characteristics of the Tablets

The bilayer tablets were fabricated using two different filaments for each part, namely, Fil-IR-PVA (50% DCNa *w*/*w* loading) for the IR layer and different filaments with the compositions presented in Table 1 for the SR layer. The obtained tablets were codified as presented in Table 2. The physical characteristics of the dosage forms were evaluated, and the obtained results are summarized in Table 3. It is noticeable that a low variability in terms of tablet dimensions was found. Additionally, the requirements of the European Pharmacopoeia regarding the “uniformity of mass of single-dose preparations” [34] were met in all cases.

### 2.5. Contact Angle Measurement

**Figure 4:**
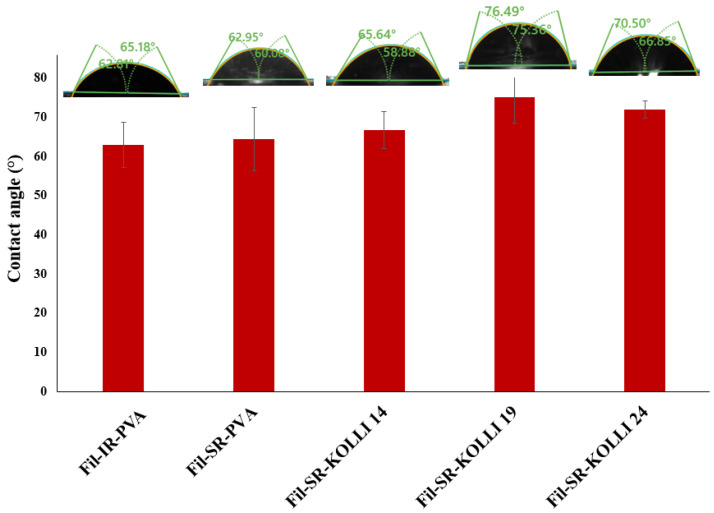
Recorded contact angles for printouts prepared with the hot-melt-extruded filaments.

The contact angle determinations provide insights into the surface properties of the materials. The results shown in Figure 4 show that all of the recorded contact angles were below 90° and thereby attest the hydrophilic nature of the samples [35,36]. Comparable contact angles were measured for the samples containing 50% *w*/*w* (based on Fil-IR-PVA) and 10% *w*/*w* DCNa (based on Fil-SR-PVA). These findings suggest that the surface properties of the samples are not influenced by drug loading. However, by including a hydrophobic polymer in the formulation, namely, Kolli SR [37], the wettability of the samples decreased as reflected by the increased contact angles recorded compared to the samples prepared with filaments lacking the hydrophobic component (Fil-IR-PVA and Fil-SR-PVA). However, significant statistical differences in terms of wettability were identified between the filament used for the preparation of the IR layer and filaments containing 19% and 24% *w*/*w* Kolli SR. Thereby, the data confirm the suitability of Kollidon^®^ SR to function as a hydrophilia-decreasing agent when included in a proportion of at least 19% *w*/*w* in the formulation, with the potential to prolong the release of the drug from the 3D-printed fragments prepared with filaments that contain it.

### 2.6. Drug Content

Ensuring a drug content as close as possible to the theoretical values is of utmost importance for pharmaceutical preparations. For drug products fabricated through HME-FDM-3DP, the drug-loading efficiency is a valuable indicator of the API stability, since two thermal processing steps are involved in the methodology, and of the homogenous distribution of the drug in the intermediate and final products. In this regard, the DCNa contents of the filament and tablet samples were investigated, and the results are displayed in Table 4. Drug contents above 92% of the theoretical values were evidenced by the determinations for both filament and tablet samples. Similar results were obtained for the formulations with higher drug loadings (50% *w*/*w*) and those with lower ones (10% *w*/*w*), suggesting that the differences in terms of polymer proportion did not affect the ability of the API to maintain its integrity during thermal processing. The TGA investigations highlighted the stability of the API at the employed working temperatures, which was further confirmed by the present evaluation. The relatively lower API-loading values in both filaments and tablets compared to the theoretical ones were presumably obtained due to the fine DCNa powder sticking to the walls of the barrel during the extrusion process, as revealed in other works reported in the literature [38]. Additionally, although the results also reveal a good homogeneity of API distribution in both intermediate and final products, the outcome could be further improved by employing HME equipment with a twin-screw configuration [39].

### 2.7. In Vitro Dissolution Behavior

**Figure 5:**
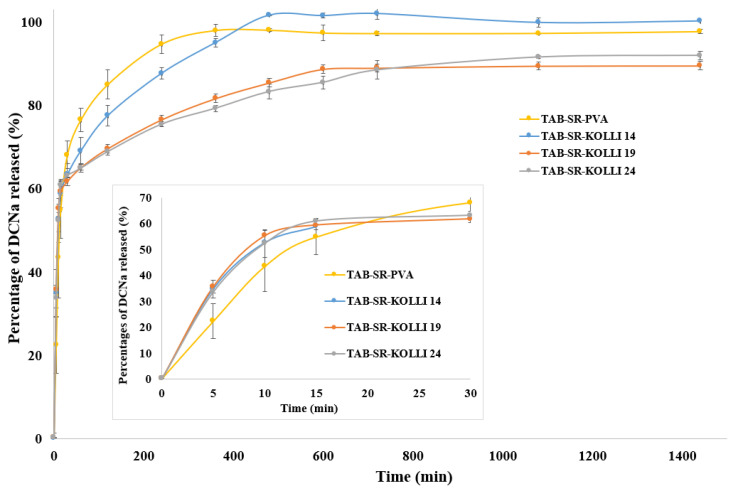
In vitro dissolution profiles of the DCNa-loaded bilayer tablets.

The in vitro dissolution profiles of the bilayer tablets prepared via FDM-3DP are displayed in Figure 5. It is noticeable that, as desired, the fast release of an initial dose of DCNa was obtained for each formulation. The API percentages released in 30 min reached 68.16% for TAB-SR-PVA, 63.4% for TAB-SR-KOLLI 14, 61.8% for TAB-SR-KOLLI 19, and 63.18% for TAB-SR-KOLLI 24. The IR layer of the tablets is primarily accountable for the rapid API dissolution since this sought-after phenomenon was ensured by applying proper tablet design and formulation strategies (i.e., a honeycomb pattern and feedstock filament loaded with 50% *w*/*w* DCNa, respectively). The presumption is also supported by the behavior of the tablets visualized during the examinations, since the fast disintegration of the IR layer was clearly distinguishable. However, the drug percentages released in 30 min were also constituted by the APIs dissolved from the SR layer, since only approximately 59% *w*/*w* of the API doses contained by each tablet were distributed in the IR layer. As opposed to the initial segments of the dissolution profiles, mainly governed by the release of DCNa from the IR layer, which were almost superimposable, a variability of the drug release rate from the SR layer was encountered. The differences depended on the qualitative and quantitative composition of the filaments used for the fabrication of the tablet section. The results reveal that the fastest dissolution was obtained for TAB-SR-PVA, the formulation based solely on PVA as a polymeric constituent. The compositions that also contained Kollidon SR exhibited slower release rates. More specifically, an increased Kollidon SR content negatively impacted the dissolution process. However, the differences observed in the dissolution behavior of the tablets with the SR layer based on PVA and those relying on the combination of PVA and Kolli SR in different ratios were not statistically significant. In addition, it is observable that, within a 24 h timeframe, approximately 90% *w*/*w* of the drug doses included in TAB-SR-KOLLI 19 and TAB-SR-KOLLI 24 were released, while the rest remained trapped in undissolved tablet fragments. Therefore, the results reflect the efficiency of the design modulation strategy (100% infill layer) in decreasing the drug release rate from the SR layer. Additionally, the higher polymeric fraction compared to the IR layer contributed to the observed effects, as suggested by the literature [17]. However, the presence of Kollidon^®^ SR in the formulation in the evaluated proportions (14%, 19%, and 24% *w*/*w*) did not significantly impact the dissolution behavior. Thus, the association of other excipients with the potential to decelerate the erosion of the polymeric matrix should be explored to further prolong the release of the drug from the SR layer.

The API release profile kinetics were investigated by fitting the dissolution data to the following mathematical models: zero-order, first-order, Korsmeyer–Peppas, Hixon and Crowell, Baker and Lonsdale, and Higuchi. The values of the calculated Akaike Indices (AIC), dissolution constants (k), and release exponents (n) are shown in the Supplementary Materials (Table S1). For all the tablets, the release data fitted well with the Korsmeyer–Peppas model, which is generally suitable to describe a porous system of a swollen polymeric matrix that sorbs the solvent and desorbs the APIs along with the dissolution of the polymeric structure [40]. The release exponent values also provide an insight into the involved drug release mechanisms. Thus, the values of n ≤ 0.45 indicate a Fickian diffusion mechanism, which implies a diffusion-controlled API release from the developed bilayer tablets [41].

## 3. Materials and Methods

### 3.1. Materials

The model drug, diclofenac sodium (DCNa), was gifted by Aarti Drugs Ltd. (Mumbai, India). D-Sorbitol and PVA Parteck^®^ MXP were purchased from Millipore Sigma (Billerica, MA, USA). Kolliphor^®^ TPGS (KTPGS) and Kollidon^®^ SR (Kolli SR) were acquired from BASF (Ludwigshafen, Germany). Aerosil^®^ (colloidal silicon dioxide) was obtained from Evonik Industries AG (Essen, Germany).

### 3.2. Filament Preparation by HME

The filaments were fabricated by processing the physical mixtures with the compositions presented in Table 1 via HME. The blends were prepared by mixing the pre-weighed components in a mortar and pestle. A single-screw equipment (Noztek Pro, Noztek, UK) with a screw speed of 65 rpm and one electrically heated compartment was employed for the fabrication of the drug-loaded filaments. The feeding rate of the mixtures was kept at 1–2 g/min. The compositions were processed at 175 °C (PM-SR-PVA, PM-SR-KOLLI 14, PM-SR-KOLLI 19, and PM-SR-KOLLI 24) and 190 °C (PM-IR-PVA), followed by extrusion through a 1.75 mm die. The filaments were collected manually and stored at room temperature in sealed plastic bags.

### 3.3. Mechanical Characterization of the Hot-Melt-Extruded Filaments

**Figure 6:**
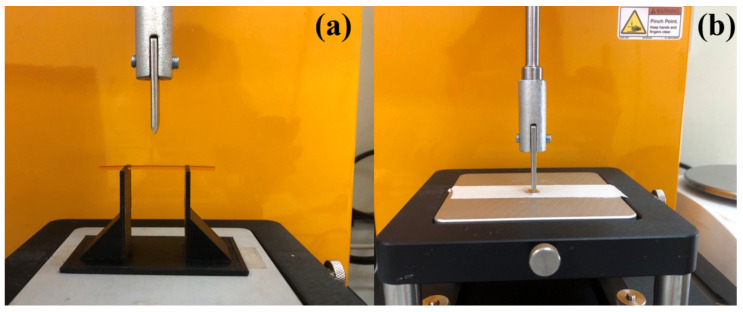
Experimental setup for the (**a**)—three point bending test and (**b**)—stiffness test.

The mechanical properties were assessed through texture analysis, performing two tests known as the Repka–Zhang tests [42]. The diameter of all samples was measured prior to the mechanical tests using a digital caliper with a resolution of 0.01 mm (Parkside, Germany). First, a 3-point bending fixture with a 25 mm gap rig and a vertical blade probe were installed on a CT3 Texture Analyzer (Brookfield Ametek, Middleboro, MA, USA) equipped with a 4.5 kg load cell. The 50 mm filament samples were placed horizontally on the rig (Figure 6a), and then the blade descended to the sample at a speed of 10 mm/s down to a distance of 15 mm. The load versus time profiles were recorded, and the maximum load and the breaking distance were determined out of five replicate measurements using TexturePro CT software V1.10 (Brookfield Ametek, Middleboro, MA, USA). Furthermore, the flexural stress, strain, and stiffness values were calculated as per Samaro et al. [43] and Hu et al. (2022) [44].

The stiffness test (Figure 6b) was performed on a CT3 Texture Analyzer (Brookfield Ametek, Middleboro, MA, USA) equipped with a 50 kg load cell. The 5 mm filament samples were placed horizontally on a flat plate, and then the blade probe descended to the sample at a 0.1 mm/s speed down to a displacement of 5%. The maximum load was determined out of the recorded load versus time profiles for 5 samples of each formulation.

### 3.4. Fabrication of the FDM-3D-Printed Bilayer Tablets and Evaluation of Their Physical Characteristics

**Figure 7:**
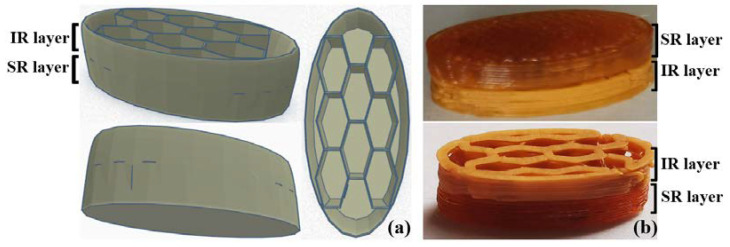
(**a**)—Images of the tablet architecture designed in TinkerCad software; (**b**)—images of the FDM-3D-printed tablets.

The architecture of the tablets was created in TinkerCad software (https://www.tinkercad.com/ Autodesk^®^ Inc., San Rafael, CA, USA). The following dimensions were established: length = 18 mm, width = 8 mm, and height = 6 mm. The design of the tablet is displayed in Figure 7a, and it is divided into two distinct layers. The first one is a honeycomb pattern surrounded by a shell, further referred to as the IR layer, while the second one is entirely filled and further referred to as the SR layer. The printing process was conducted using a MakerBot Replicator 2X (MakerBot, Brooklyn, NY, USA) fed with a different filament for each of the two halves. The honeycomb layer was invariably fabricated using the filament Fil-IR-PVA. The layer with 100% infill was prepared using Fil-SR-PVA, Fil-SR-KOLLI 14, Fil-SR-KOLLI 19, or Fil-SR-KOLLI 24. The codification of the manufactured dosage forms (Figure 7b) is presented in Table 2. The printing process was performed by the deposition of layers of 0.3 mm. Other working parameters included the printing temperature being set at 185–190 °C, platform temperature at 45 °C, first-layer print speed at 30 mm/s, and infill print speed at 90 mm/s.

Three tablets were selected randomly from each batch and their dimensions were manually measured using a Parkside IAN380693 digital caliper (Parkside, Neckarsulm, Germany) with a 0.01 mm resolution. The determination of the weight uniformity was conducted on four units of each tablet type by weighing with a digital balance (Ohaus^®^ Analytical Plus balance) and calculating the average weight and deviation from the average mass.

### 3.5. Solid State Evaluations

#### 3.5.1. Thermogravimetric Analysis

Thermogravimetric evaluations were performed using a TGA SDTA 851e thermobalance (Mettler Toledo GmbH, Zurich, Switzerland). The APIs, excipients, physical mixtures, drug-loaded filaments, and 3D-printed tablet samples weighing approximately 5 mg were each placed in 70 µL open alumina pans. The TGA curves were obtained by submitting the samples to heating in the temperature range of 25–500 °C, with a heating rate of 10 °C/min. N_2_ was used as an inert purge gas (50 mL/min). The obtained data were analyzed via Mettler Toledo STAR SW 12.10 software to evaluate the thermal decomposition of the samples.

#### 3.5.2. Differential Scanning Calorimetry

The DSC examinations were conducted using a DSC 822 equipment (Mettler Toledo GmbH, Zurich, Switzerland). The accurately weighed (2–3 mg) samples of the model drug, excipients, physical mixtures, drug-loaded filaments, and 3D-printed tablets were placed into 40 µL aluminum pans and closed with pierced lids. The samples were subjected to heating with a rate of 10 °C/min within the temperature range of 25–400 °C. Data were collected with Mettler Toledo STAR SW 12.10 software and evaluated to identify the physical state conversions that might occur during the fabrication steps.

#### 3.5.3. X-ray Diffraction

Samples of the APIs, excipients, physical mixtures, filaments, and 3D-printed tablets were subjected to XRD analysis by employing a Bruker D8 Advance powder diffractometer (Bruker AXS GmbH, Karlsruhe, Germany) with Cu Kα1 radiation and the X-ray tube operating at 40 kV and 40 mA. The diffractometer was equipped with a germanium monochromator, which is used in order to obtain only the Cu-Kα1 radiation and a LINXEYE detector. All samples were evaluated with DIFFRAC plus the XRD Commander Software considering an angular range of 2°–85° 2θ and a scan speed of 0.02 θ/s.

#### 3.5.4. Fourier-Transform Infrared Spectroscopy

FTIR evaluations were conducted by mixing the powdered samples with KBr to obtain thin pellets with a thickness of 1.5 mm and a weight ratio of sample:KBr of 1:100. The spectra were recorded at room temperature in the 350–4000 cm^−1^ range on a 6100 Jasco spectrometer with a resolution of 0.5 cm^−1^ and a signal/noise ratio 42.000:1.

### 3.6. Contact Angle Measurements

The contact angle measurements were performed using a DataPhysics OCA 25 contact-angle analyzer (DataPhysics Instruments GmbH, Filderstadt, Germany) provided with an optical component that allows the visualization and recording of the interaction of the droplet with the sample and the determination of the contact angle. Investigations were conducted on samples represented by FDM-3D-printed disks (diameter = 4 cm, height = 1.5 mm) prepared with 100% infill settings. The samples were prepared using each type of fabricated filament (Fil-IR-PVA, Fil-SR-PVA, Fil-SR-KOLLI 14, Fil-SR-KOLLI 19, and Fil-SR-KOLLI 24). The wetting angles of the printlets were assessed after droplets of 2 μL of distilled water were deposited on the surfaces of the samples using the Sessile Drop method. Four examinations were performed on different regions of each sample at room temperature.

### 3.7. Drug Content Analysis

The determination of the DCNa content in filaments and 3D-printed tablets involved the dissolution of samples weighing around 37.5 mg in volumetric flasks (25 mL) filled with a mixture of methanol and water (70:30), placed in a thermostatic bath (37 ± 0.5 °C). Repeated cycles of shaking and ultrasonication (Transsonic T700, Elma, Germany) were applied to facilitate the complete dissolution of the fragments. Furthermore, samples from the dissolution medium (500 µL) were withdrawn and placed in 10 mL volumetric flasks completed to volume with phosphate buffer at pH 6.8. The obtained solutions were filtered using 0.2 µm filters (Phenomenex, Torrance, CA, USA). The API contents were evaluated by means of UV-VIS spectrophotometry at 276 nm (Specord^®^200 Plus, Analytik Jena, Jena, Germany), using a calibration curve equation that resulted from the analysis of DCNa in phosphate buffer at pH 6.8.

### 3.8. Dissolution Studies

A PT-DT70 apparatus (Pharma Test Apparatebau AG, Hainburg, Germany) with a USP type II (paddle) configuration was employed to examine the release of DCNa from the 3D-printed bilayer tablets. Each sample was placed in 900 mL phosphate buffer at pH 6.8, maintained at 37 ± 0.5 °C. The paddle rotation speed was kept constant at 50 rpm. The dissolution process was investigated for 24 h. Aliquots were withdrawn at pre-established time points (5 min, 10 min, 15 min, 30 min, 1 h, 2 h, 4 h, 6 h, 8 h, 10 h, 12 h, 18 h, and 24 h) and substituted with equal volumes of the fresh medium. The amounts of DCNa in the collected and filtered aliquots were determined spectrophotometrically (Specord^®^200 Plus, AnalytikJena, Germany) at 276 nm wavelength. Experiments were conducted in triplicate.

## 4. Conclusions

The present study provided further evidence that the flexibility of FDM-3DP enables the preparation of dosage forms with customizable characteristics. It was demonstrated that, through proper formulation and design strategies, the fabrication of tablets with a sustained API release is achievable. A novel bilayer tablet architecture was designed, consisting of an IR layer with an internal honeycomb structure intended to allow the fast release of an API dose, and a SR layer with low porosity ensured by the entirely filled configuration, which was considered to allow a slow release of the included drug. The printing was conducted via dual extrusion since an optimized filament (Fil-IR-PVA) was used for the fabrication of the IR fragment, while different feedstock filaments were evaluated for the preparation of the SR fragment. As expected, nearly superimposable dissolution patterns were obtained in the initial phase of the process, while a slow API release from the SR layer was promoted by the employed design strategy and the high polymer content. Therefore, the study demonstrated that, by the proper association of design strategies (i.e., bilayer tablet design) and formulation considerations (i.e., high polymeric fraction), the quality attributes of the final products are tunable in order to achieve the desired outcome (i.e., sustained API release). In addition, the potential of PVA to act as a versatile matrix-forming compound in both immediate- and slow-release layers was highlighted, thereby increasing the implementability of the fabrication technique in various settings by downsizing the number of materials required to obtain pharmaceutical products with diverse characteristics.

## Figures and Tables

**Table 1:** HME blend compositions and the coding of the fabricated filaments.

Blend Name	DCNa (%)	PVA (%)	Sorbitol (%)	Aerosil (%)	Kolli SR (%)	KTPGS (%)	Filament
PM-SR-PVA	10	80	9	1	-	-	Fil-SR-PVA
PM-SR-KOLLI 14	10	75	-	1	14	-	Fil-SR-KOLLI 14
PM-SR-KOLLI 19	10	70	-	1	19	-	Fil-SR-KOLLI 19
PM-SR-KOLLI 24	10	65	-	1	24	-	Fil-SR-KOLLI 24
PM-IR-PVA	50	40	-	1	-	9	Fil-IR-PVA

**Table 2:** Codification of the tablets prepared with different feedstock filaments.

IR Layer	SR Layer	FDM-3D Printed Tablet
Fil-IR-PVA	Fil-SR-PVA	Tab-SR-PVA
Fil-IR-PVA	Fil-SR-KOLLI 14	Tab-SR-KOLLI 14
Fil-IR-PVA	Fil-SR-KOLLI 19	Tab-SR-KOLLI 19
Fil-IR-PVA	Fil-SR-KOLLI 24	Tab-SR-KOLLI 24

**Table 3:** Overview of the 3D-printed tablet dimensions, average weight, and deviation from the average weight.

Tablet	Dimensions			AW ± SD (mg), n = 4	DAW (%)
	L (mm)	W (mm)	H (mm)		
TAB-SR-PVA	18.01 ± 0.20	8.13 ± 0.06	6.00 ± 0.06	740.19 ± 14.1	+1.62 −2.40
TAB-SR-KOLLI 14	17.93 ± 0.12	8.08 ± 0.06	5.97 ± 0.08	731.93 ± 9.06	+1.74 −1.14
TAB-SR-KOLLI 19	18.05 ± 0.12	8.08 ± 0.08	6.00 ± 0.03	695.51 ± 3.38	+0.46 −0.48
TAB-SR-KOLLI 24	18.02 ± 0.07	7.97 ± 0.05	6.03 ± 0.04	702.77 ± 2.29	+0.19 −0.49

L = length; W = width; H = height; AW = average weight; DAW = deviation from the average weight.

**Table 4:** Drug loadings of the filaments and tablets and the calculated yield values.

Sample	Drug Loading ± SD (%)	Yield ± SD (%)
Fil-IR-PVA	47.5 ± 0.53	95.1 ± 1.06
Tab-IR-PVA	46.8 ± 0.53	93.6 ± 0.64
Fil-SR-PVA	9.58 ± 0.2	95.8 ± 2.02
Tab-SR-PVA	9.46 ± 0.04	94.6 ± 0.37
Fil-SR-KOLLI 14	9.44 ± 0.05	94.4 ± 0.46
Tab-SR-KOLLI 14	9.22 ± 0.11	92.2 ± 1.08
Fil-SR-KOLLI 19	9.37 ± 0.06	93.7 ± 0.58
Tab-SR-KOLLI 19	9.28 ± 0.19	92.8 ± 1.91
Fil-SR-KOLLI 24	9.68 ± 0.13	96.8 ± 1.27
Tab-SR-KOLLI 24	9.43 ± 0.22	94.3 ± 2.24

## Data Availability

The data presented in this study are available on request from the corresponding author.

## Supplementary Materials

The following supporting information can be downloaded at: https://www.mdpi.com/article/10.3390/ph16091321/s1. Figure S1: TGA profiles of materials along with physical mixtures, filaments and tablets consisting of (a)—PVA or (b)—combination of PVA and 24% *w*/*w* Kollidon^®^ SR as matrix forming polymers; Figure S2: TGA profiles of raw materials along with physical mixtures, filaments, and tablets consisting of PVA and 14% *w*/*w* Kollidon^®^ SR as matrix forming polymers; Figure S3: TGA profiles of raw materials along with physical mixtures, filaments, and tablets consisting of PVA and 19% *w*/*w* Kollidon^®^ SR as matrix forming polymers; Figure S4: DSC curves of materials, physical mixtures, filaments, and tablets consisting of (a)—PVA or (b)—combination of PVA and 24% *w*/*w* Kollidon^®^ SR as matrix forming polymers; Figure S5: DSC curves of raw materials, physical mixtures, filaments, and tablets consisting of PVA and 14% *w*/*w* Kollidon^®^ SR as matrix forming polymers; Figure S6: DSC curves of materials, physical mixtures, filaments, and tablets consisting of PVA and 19% *w*/*w* Kollidon^®^ SR as matrix forming polymers; Figure S7: XRD diffractograms of materials, physical mixtures, filaments and tablets consisting of combinations of PVA and Kollidon^®^ SR in different ratios as matrix forming polymers; Table S1: Results obtained after fitting the drug release data to different mathematical models.
