# Supplementary material for: Development of a Bilayer Tablet by Fused Deposition Modeling as a Sustained-Release Drug Delivery System
Source: Pharmaceuticals (Basel). 2023 Sep 19;16(9):1321. doi: 10.3390/ph16091321 (PMC10537489; doi:10.3390/ph16091321)
Supplement: Supplementary file 1 [file pharmaceuticals-16-01321-s001.zip › pharmaceuticals-2557146-supplementary.pdf]

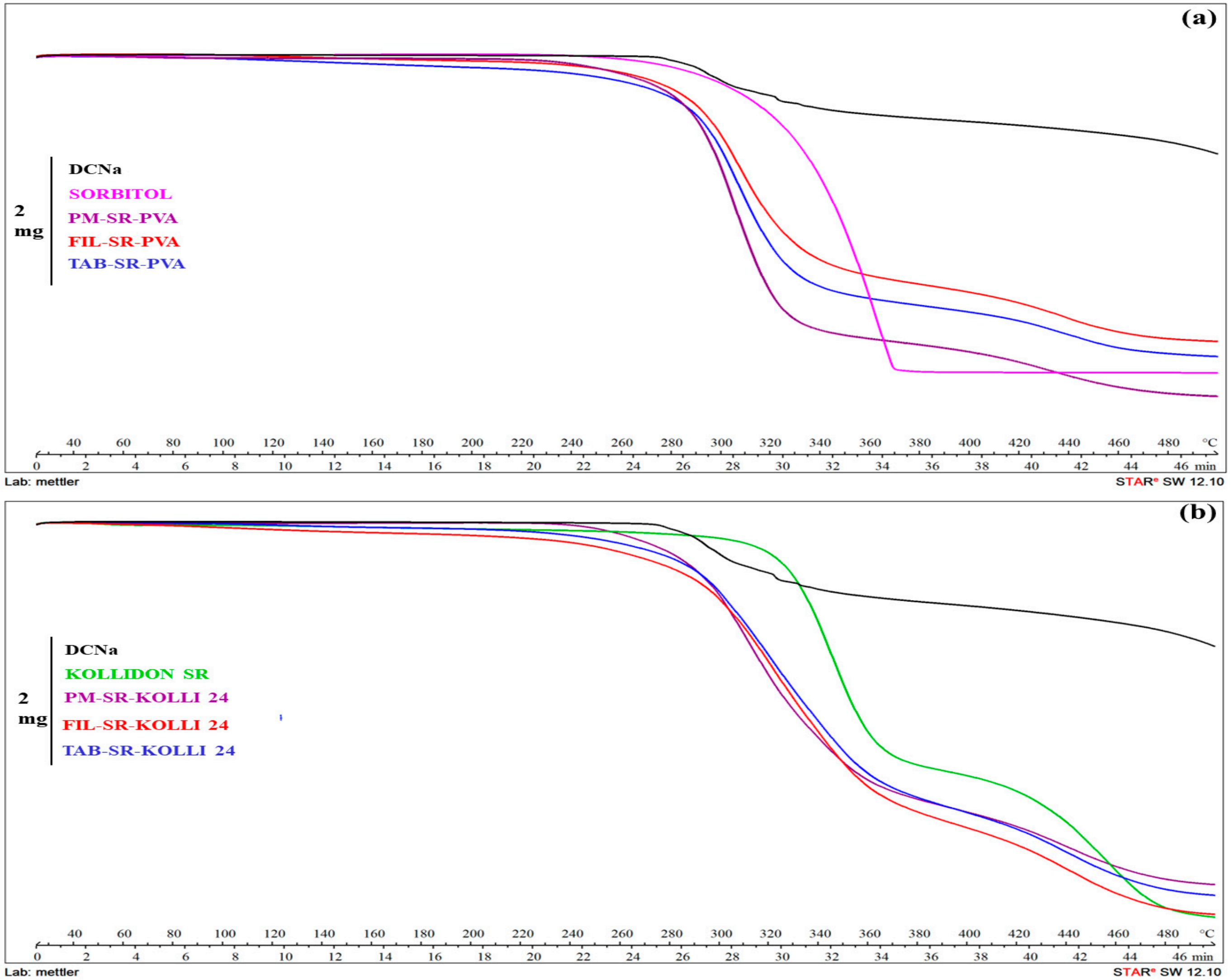

Figure S1. TGA profiles of materials along with physical mixtures, filaments and tablets consisting of (a) – PVA or (b) – combination of PVA and 24% w/w Kollidon® SR as matrix forming polymers.

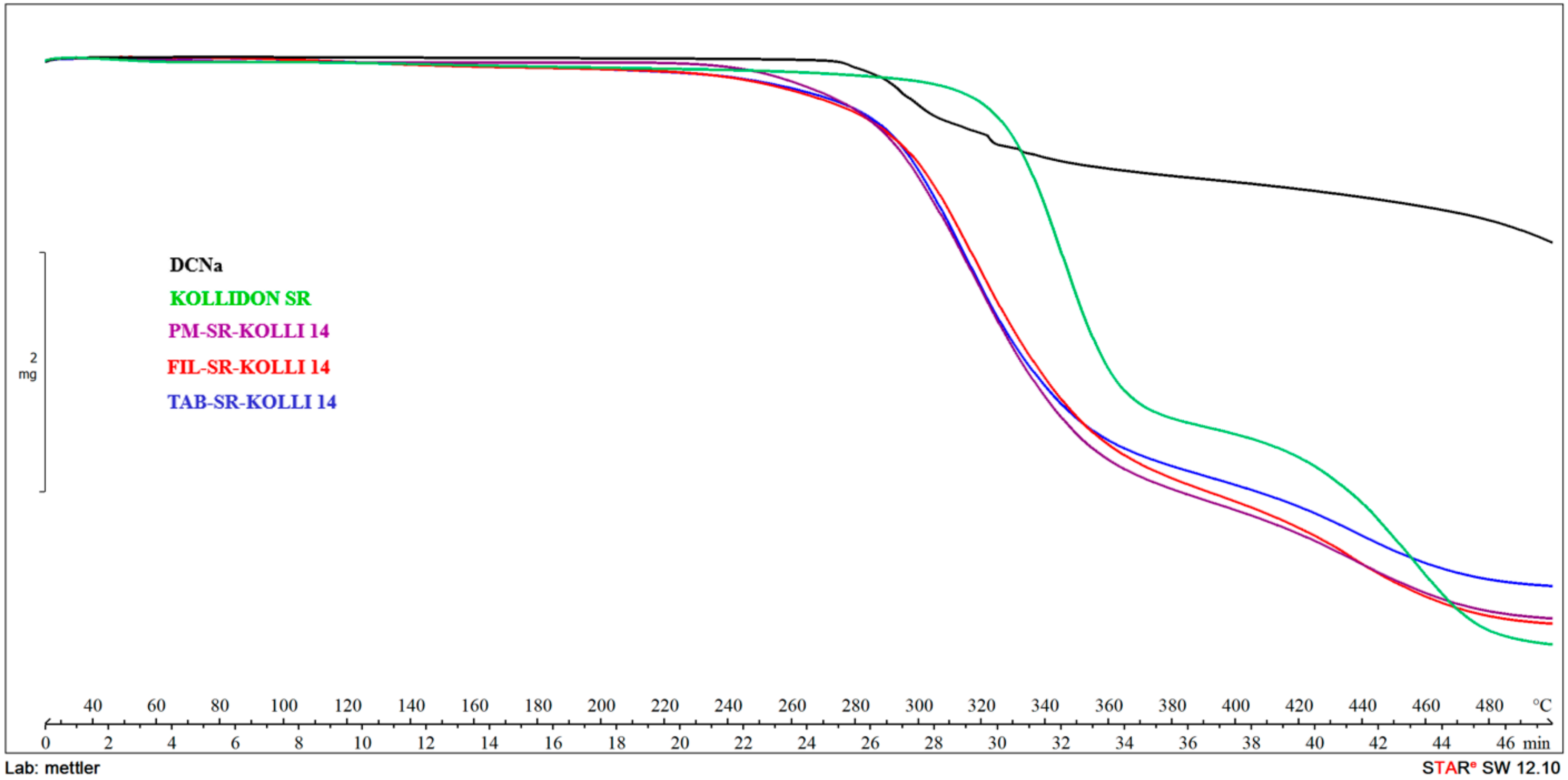

Figure S2. TGA profiles of raw materials along with physical mixtures, filaments, and tablets consisting of PVA and 14% w/w Kollidon® SR as matrix forming polymers.

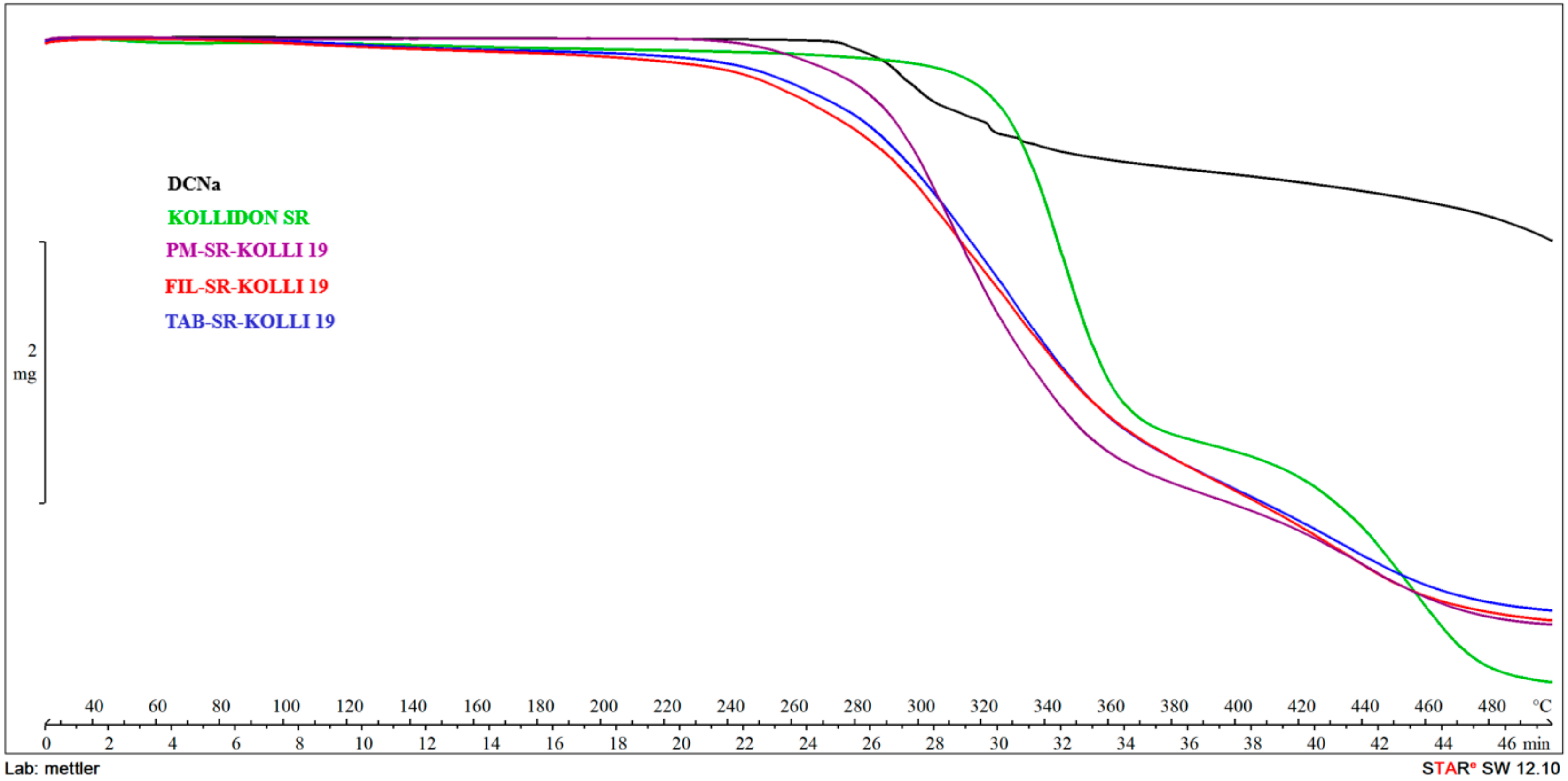

Figure S3. TGA profiles of raw materials along with physical mixtures, filaments, and tablets consisting of PVA and 19% w/w Kollidon® SR as matrix forming polymers.

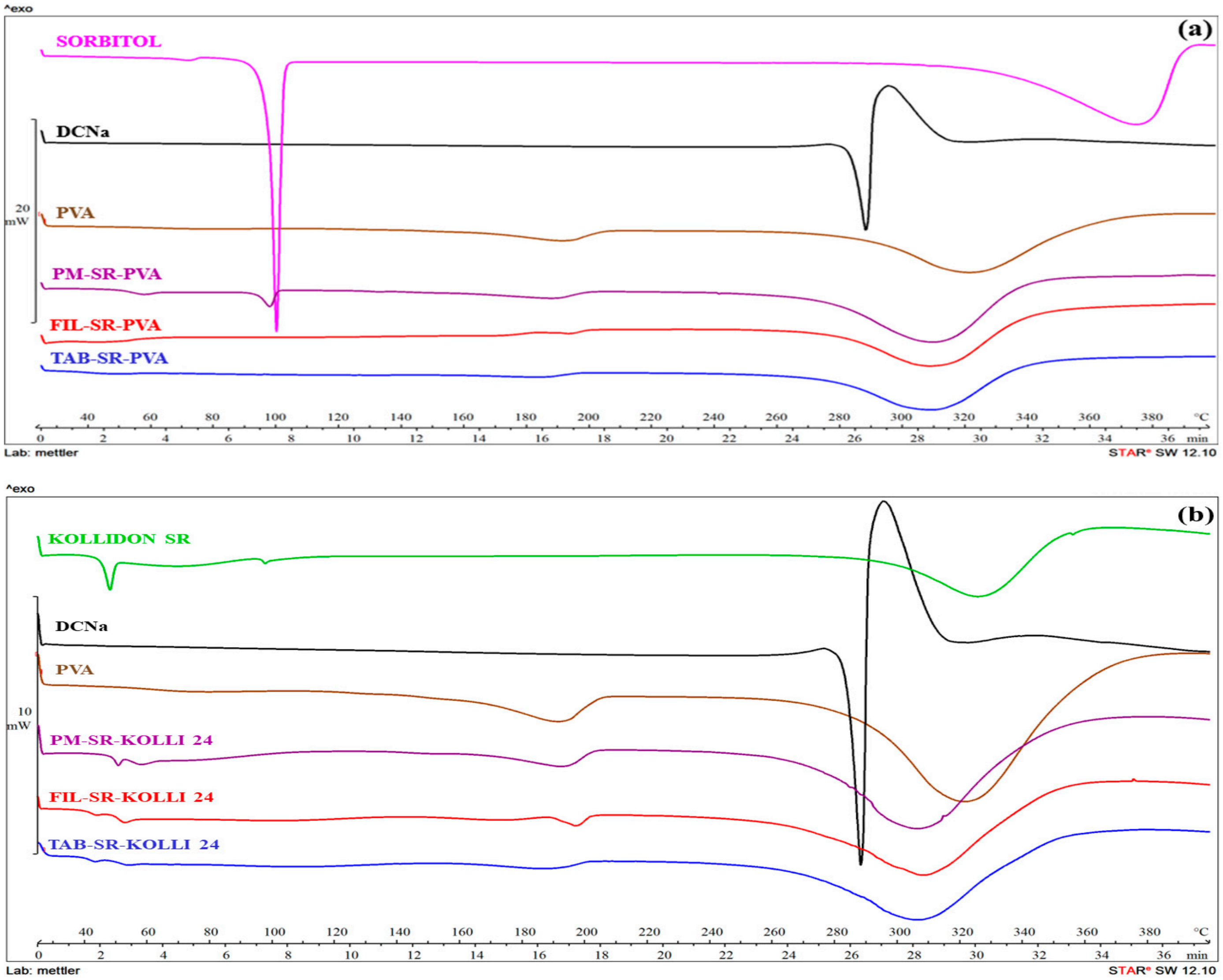

Figure S4. DSC curves of materials, physical mixtures, filaments, and tablets consisting of (a) – PVA or (b) – combination of PVA and 24% w/w Kollidon® SR as matrix forming polymers.

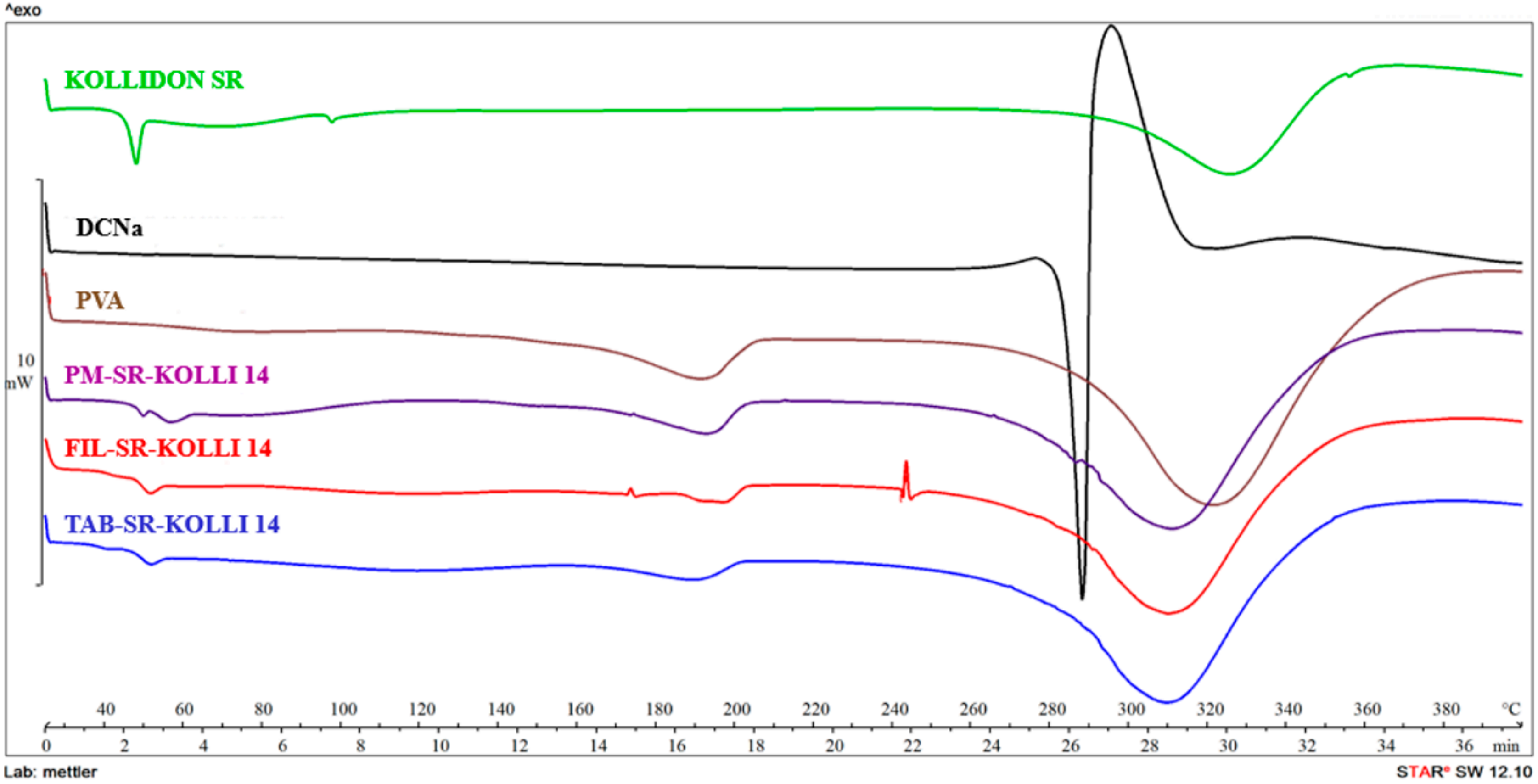

Figure S5. DSC curves of raw materials, physical mixtures, filaments, and tablets consisting of PVA and 14% w/w Kollidon® SR as matrix forming polymers.

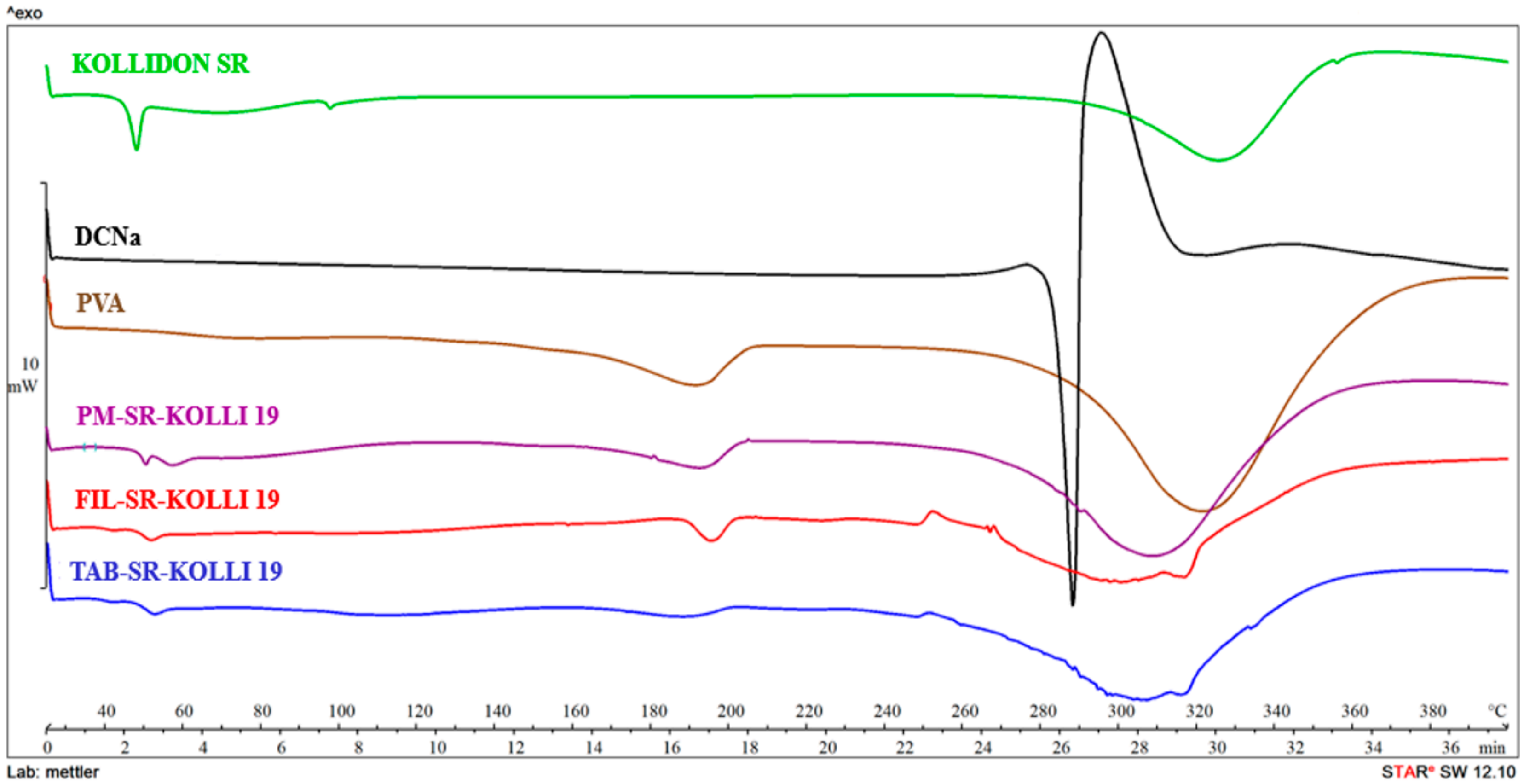

Figure S6. DSC curves of materials, physical mixtures, filaments, and tablets consisting of PVA and 19% w/w Kollidon® SR as matrix forming polymers.

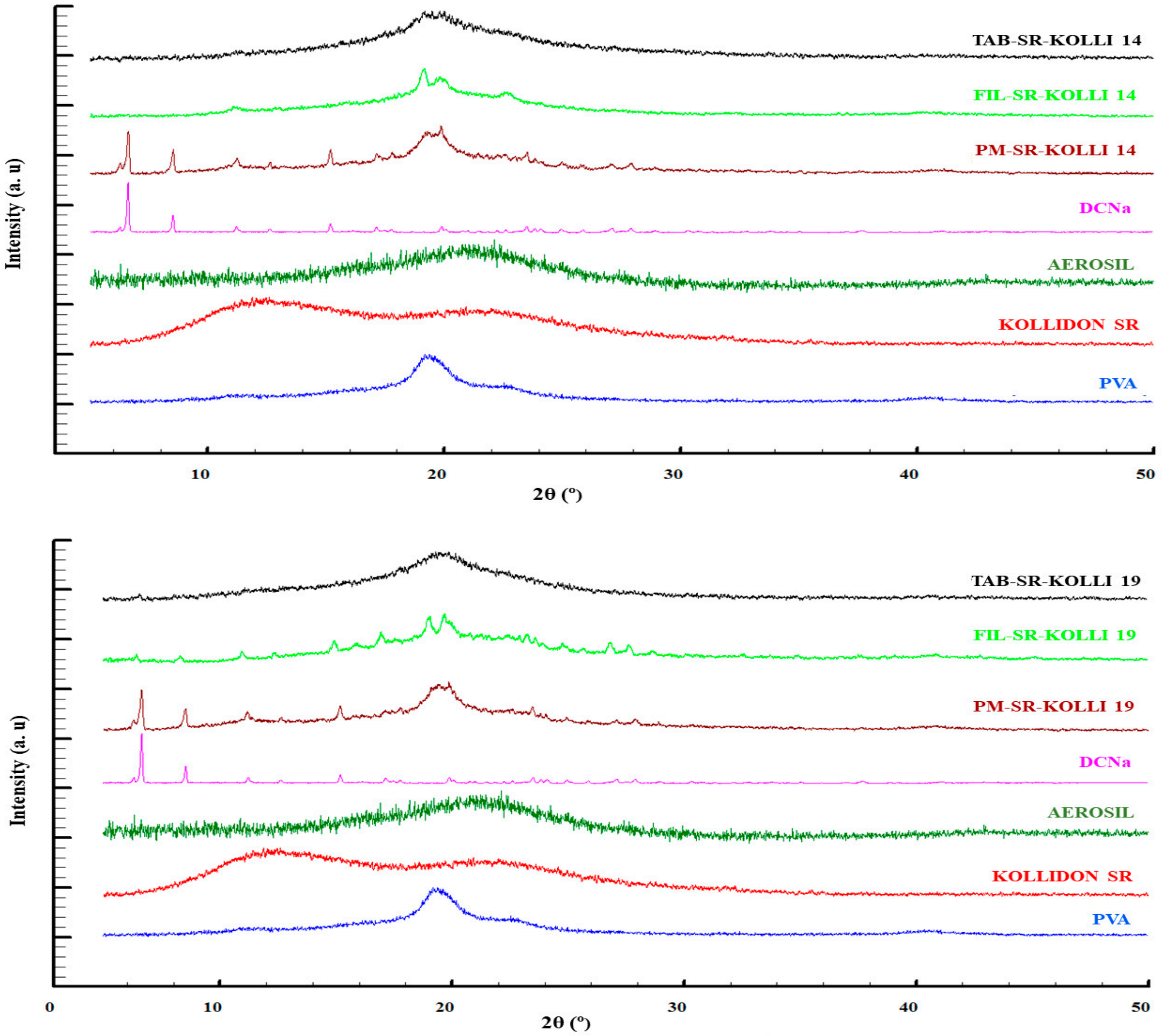

Figure S7. XRD diffractograms of materials, physical mixtures, filaments and tablets consisting of combinations of PVA and Kollidon® SR in different ratios as matrix forming polymers.

Table S1. Results obtained after fitting the drug release data to different mathematical models.

Formulation	Zero order		First order		Higuchi		Baker and Lonsdale		Hixon and Crowell		Korsmeyer-Peppas		
	AIC	k (%min^{-1})	AIC	k (min^{-1})	AIC	k (%min$^{-1/2}$)	AIC	k (%$^{2/3}$min^{-1})	AIC	k (%$^{1/3}$ min^{-1})	AIC	K (%min^{-n})	n
**TAB-SR-PVA**	66.7	0.533	47.3	0.0436	57.0	7.84	43.2	0.0027	57.3	0.0062	46.2	25.00	0.254
**TAB-SR-KOLLI 14**	78.2	0.350	62.5	0.0497	69.7	6.39	60.7	0.0018	71.3	0.0042	42.6	32.91	0.181
**TAB-SR-KOLLI 19**	88.9	0.239	75.8	0.0494	81.2	5.07	72.2	0.0023	83.4	0.0030	50.5	37.41	0.134
**TAB-SR-KOLLI 24**	110.2	0.165	93.5	0.0481	101.4	4.19	93.5	0.0009	104.0	0.0021	64.5	37.35	0.131

AIC = Akaike Information Criterion, k = dissolution constant, n = release exponent (indicative of drug release mechanism).
